# The use of Method Information Index (MII) to monitor the amount of information given to women users of modern contraceptives in Indonesia: results from an analysis of the 2007, 2012 and 2017 demographic and health surveys

**DOI:** 10.1186/s12905-022-02094-1

**Published:** 2022-12-02

**Authors:** Meiwita P. Budiharsana, Wiji Wahyuningsih, Peter Heywood

**Affiliations:** 1 Population and Biostatistics Department, Faculty of Public Health, Universitas Indonesia, Depok, Indonesia; 2 Menzies Centre for Health Policy, University of Sydney, Sydney, NSW Australia

**Keywords:** Methods Information Index (MII), Information received, Modern contraceptives, Indonesia, 2007–2017

## Abstract

**Background:**

The use of Method Information Index (MII) indicates whether women contraceptive users receive adequate information about all available contraceptive methods, side effects of the methods, and how to deal with the side effects if experienced—at method initiation.

**Objective:**

This study aims to investigate the level of MII scores or the amount of information received by married women users of five modern contraceptives at the time of initiation and changes of its determinants based on the Indonesian Demographic and Health data between 2007 and 2017.

**Methods:**

Data of married women who used most common five modern contraceptive methods (the pill, injectables, implants, IUD, and female sterilization), comprised of a total unweighted sample of 35,412 users out of the 32,895; 45,607 and 49,627 women aged 15–49 in the 2007, 2012, and 2017 Indonesian Demographic and Health Survey (IDHS), respectively. The Method Information Index (MII) scores were calculated based on responses to three questions (whether women were told about method‐specific side effects, advised what to do if they experienced them, and informed about other available methods). Multivariable logistic regressions with ‘time’ as an interaction variable were used to assess the influence of time upon the MII scores and its determinants.

**Results:**

The MII scores were 23.84% in 2007, 24.60% in 2012 and 28.65% in 2017. Obviously, over 70% of reproductive-age women contraceptive users were not receiving complete information about modern contraceptives at the time of initiation. After 5 years (2012), only living in the Java Bali region (AOR = 1.34, 95% CI 1.09–1.66) compared to living in other islands, and currently using injectables (AOR = 1.43, 95% CI 1.10–1.87) and currently using implants (AOR = 1.68, 95% CI 1.07–2.63) compared to currently using pills had significantly higher odds of receiving MII information. After 10 years (2017), only one variable (the ‘richest’ in the wealth quintile category (AOR = 0.70, 95% CI 0.50–0.99) compared to the ‘poorest’) still showed a significant association with receipt of complete MII information.

**Conclusions:**

Despite the fact that the MII scores increased gradually across the years, interaction with ‘survey time’ showed that the likelihood of receiving complete MII information was not statistically different in the 5 years (2007–2012) and in the 10 years (2007–2017) period from the reference category in 2007. The authors recommend use of the MII score as an objective measure to evaluate access to MII essential information and to monitor an increase in the informed population in Indonesia.

## Introduction

Indonesia was one of the countries that participated in the 2012 London Summit on Family Planning (FP) meeting. At that meeting, participating countries committed to make a sustaining coverage of modern methods of contraception for an additional 120 million women and girls in the world’s poorest countries by 2020. Access to modern contraceptives will open opportunities for women to participate in and support their communities’ social and economic development [1]. Indonesia has also declared a commitment to reach universal access to sexual and reproductive health-care services, including for family planning information and education, in its national health strategies and programmes by 2030. The National Population and Family Planning Program is the responsible ministry for monitoring the access and quality of FP information and education programs. This study aims to assess the level of the Method Information Index (MII) in Indonesia, the extent to which it has changed over the decade between 2007 and 2017.

Until recently, modern methods accounted for about 90% of contraceptives use. Among the five modern contraceptive methods (the pill, injectables, implants, IUD, and female sterilization), the pill and injectables accounted are dominant. The use of long-term contraceptive methods remains low in the last two decades. Literature showed that reasons to choose short-acting rather than long-acting methods are lack of knowledge about and fear of side-effects of long-term contraceptive methods [2]. Studies also point to the association between the Village Midwife Program in Indonesia and increased injectable contraceptives used [3, 4].

Increasing knowledge about long-term contraceptive methods among women and adolescent girls are crucial for reducing the fear of side effects, myths about causing infertility, and other misleading rumours [2, 5]. Nonetheless, the National Population and Family Planning Program changed its priorities to place more emphasis on family welfare and stunting [6]. Considering that FP information and services are essential in improving the health and rights of women in Indonesia, stronger family planning information and education strategies are needed, including improving the capacity of midwives and other trained health care providers to give complete information on all available modern contraceptives to minimize the fear about adverse effects and misinformation [7].

Receiving complete information increases clients’ confidence in and commitment to their decisions about the contraceptive they choose [8, 9]. Conversely, receiving inadequate information about the side effects of a currently used method contributes to high discontinuity rates. The effect of incomplete information can also be reflected in another indicator, the discontinuation rate of using a method. The 2017 IDHS data revealed that the primary reason for discontinuation was fear of the unknown about side effects (33.2%) [10]. This study examines, as reflected in the MII, the proportion of Indonesian women contraceptive users who received information about method‐specific side effects, what to do if they experienced them, and information about other available methods among the five modern contraceptive methods (female sterilization, implants, injections, IUD, and the pill) in the 2007, 2012 and 2017 IDHS datasets.

## Methods

### Data sources

The study used secondary data analysis of the individual records of 2007, 2012, and 2017 Indonesia Demographic and Health Survey (IDHS) data. Under the US Agency for International Development financial support, all three IDHS data sets are publicly available at https://dhsprogram.com/data/available-datasets.cfm,. Verbal informed consent was obtained from all respondents by IDHS, who also removed all identifiers, which allowed this study to be implemented without additional ethic approval.

### Study design, sample size, and participants

The IDHS is a large-scale cross-sectional household survey that collects information from nationally representative samples, follows an international methodological approach, and is conducted every 5 years. These repeated cross-sectional surveys employed a multistage stratified sampling design to generate a nationally representative sample from all 34 provinces in Indonesia. A total of 32,895, 45,607 and 49,627 women aged 15–49 completed the IDHS interviews with a response rate of 96.0% (2007), 95.9% (2012) and 97.8% (2017), respectively [10]. The unit of analysis in this study was women aged 15–49 years who were married/living together with a partner and started the last episode of use of any of five modern contraceptives (female sterilization, pill, IUD, injectables, and implants) within the 5 years preceding each survey (2007, 2012 and 2017 IDHS). Non-contraceptive users and users of methods other than the five modern contraceptives listed were excluded due to the fact that questions on informed choices were only asked to women users of these five contraceptive methods. These five modern contraceptives accounted for 97.4% (2007), 90.2% (2012) and 85.0% (2017) of method mix (and additional methods that were not being asked in the method mix questions). We undertook analysis of married women only because the number of non-married women reporting using modern contraceptive methods was very small. This study included 11,502 women from IDHS 2007; 12,029 women from IDHS 2012; and 11,881 women from IDHS 2017 datasets.

### Study variables

The dependent variable in this study was the reported value for Method Information Index (MII) scores, which was derived from the number of current women users of the five selected modern contraceptive methods who received all three-essential information on side effects, what to do when they experienced side effects, and other available methods as alternatives at the initiation of the usage [11, 12]. The numerators were the number of women who received all three (MII) information at the start of the current episode of using the method in each survey. The denominators were the number of women contraceptive users of all the five modern methods of pills, IUDs, injectables, implants, and female sterilization in each survey [13]. The MII scores were calculated using the answers to these three essential questions: (1) “Were you informed about possible side effects or problems you might have with the method?”; (2) “Were you told what to do if you experience any side effects or problems?”; and (3) “Were you informed about other methods of family planning?” [14]. Answers were coded as 1 = yes or receiving all these three pieces of information, and 0 = not receiving all three pieces of information. The independent variables included selected sociodemographic factors (age of respondents, education attainment, women’s occupation, wealth quintile, residency, region), exposure to radio, television, and newspapers, parity, source of FP methods, current contraceptive method use, and knowledge of all five modern contraception methods (female sterilization, pill, injectables, IUD, and implants).

### Statistical analysis

The univariate analysis shows the frequency distribution of each selected variable in all three waves of IDHS (2007, 2012, and 2017) datasets. Multivariable logistic regression was applied with MII score as our dependent variable (value = 0-not receiving all three MII information; value = 1-receiving all three MII information). In the logistic regression analysis, odds ratio (COR), adjusted odds ratio (AOR), standard errors (SE) and 95% confidence intervals (CI) were used to measure the strength of association between independent variables and the dependent variable. The final multivariable logistic model included all variables. To assess the effect of time on the MII scores, we added a dummy ‘time’ variable for 2012 and 2017, while 2007 was used as the baseline or reference. The 2012 data represented the effect of time after 5 years, and the 2017 data as the effect of time after 10 years [15]. Multivariable logistic regression analysis with time interaction was used to see whether time as a third variable influenced the relationship between each of the independent variables and the dependent variable (scores of receiving complete MII information).

The IDHS data employed individual-level sampling weights to produce nationally representative results. All subjects were weighted by their sampling probability [16]. Data management and analysis were performed using STATA version 16.0 (Stata Corporation, USA). The effect of survey estimation [svy] command in STATA was also used to adjust for the complex survey design characteristics.

## Results

After removing respondents with missing values on selected study variables, the total unweighted sample size for the 2007 IDHS dataset was 11,502 users of the five modern contraceptives; the 2012 IDHS dataset was 12,029 users of the five modern contraceptives, and the 2017 IDHS 11,881 users of the five modern contraceptives. The overall MII values of users of the five modern contraceptives who received all three MII information in Indonesia were 23.84% in 2007, 24.60% in 2012, and 28.65% in 2017 (see Table 1), based on those who responded “yes” to all three questions [14]. Individual responses when asked whether received information about side effects was 39.41% in 2007, 40.66% in 2012, and 44.18% in 2017. The lowest responses were for whether women received information on what to do when experiencing side effects, only 37.01% in 2007, 29.54% in 2012, and 34.04% in 2017. The highest individual responses were for whether women received information about other available methods, 47.97% in 2007, 58.02% in 2012, and 62.05% in 2017.

**Table 1:** Percentages of women contraceptive users receiving information about modern contraceptives at the time of initiation in 2007, 2012 and 2017

	2007 IDHS	2012 IDHS	2017 IDHS
	n (%)	n (%)	n (%)
Received information on side effects or problems of the method used	4563 (39.41)	4512 (40.66)	5090 (44.18)
Received information on what to do if experienced side effects or problems with the method used	4197 (37.01)	3282 (29.54)	3870 (34.04)
Received information on other methods of contraception that could be used	5661 (47.97)	6710 (58.02)	7301 (62.05)
Received information on all three above (MII scores)^a^	2823 (23.84)	2722 (24.60)	3271 (28.65)

^a^Information about side effects of the methods used, what to do if they experienced side effects or problems of the methods used and other available methods of contraception that could be used

Table 2 shows the frequency distribution of socio-demographic characteristics of married women using the five modern contraceptives included in the three surveys. The percentage of married women 15–24 years using the five modern contraceptives (16.11%) was the lowest in 2017 compared to 22.64% in 2007 and 20.22% in 2012. Across the three surveys, the proportion of women with secondary and higher education increased; and the proportion of primary and no education decreased. Over 45 percent of contraceptive users were unemployed in all three surveys. Most of the women contraceptive users were in the middle and rich household wealth quintiles categories (41.62% in 2007; 43.29% in 2012, and 42.72% in 2017). Respondents were more distributed in the rural areas (59.14% in 2007; 52.65% in 2012; and 54.72% in 2017). Over 60 percent of women users resided in the Java-Bali region in all three surveys.

**Table 2:** Proportions of socio-demographic characteristics of married women using the five modern contraceptives, according to the 2007, 2012 and 2017 IDHS datasets

Characteristics	2007	2012	2017
	N1 = 11,502 (%)	N2 = 12,029 (%)	N3 = 11,881 (%)
*Age*			
15–24 years	22.64	20.22	16.11
25–34 years	48.38	47.40	43.86
35–49 years	28.98	32.38	40.03
*Education*^*a*^			
No education	2.69	1.56	0.82
Primary	43.73	37.18	32.67
Secondary	47.18	53.23	56.90
Higher education	6.41	8.02	9.61
*Occupation*			
Unemployed	47.98	44.79	46.67
Agriculture	19.45	11.89	11.84
Blue-collar	29.05	38.01	36.12
White-collar	3.51	5.31	5.37
*Wealth quintiles*			
Poorest	18.16	18.15	19.35
Poor	21.44	21.00	21.90
Middle	20.94	21.47	21.70
Rich	20.68	21.82	21.02
Richest	18.77	17.56	16.03
*Residence*			
Rural	59.14	52.65	54.72
Urban	40.86	47.35	45.28
*Region*			
Other islands	36.91	38.07	39.03
Java-Bali	63.09	61.93	60.97
*Heard about FP on radio in the last few months*			
No	90.08	48.63	39.24
Yes	9.92	51.37	60.76
*Heard about FP on television in the last few months*			
No	71.29	51.94	40.54
Yes	28.71	48.06	59.46
*Read about FP in a newspaper in the last few months*			
No	87.66	86.99	89.44
Yes	12.34	13.01	10.56
*Parity*			
0–1	32.77	33.42	27.82
2–3	50.95	52.42	59.61
4 +	16.28	14.16	12.57
*Source of FP methods*			
Midwives^b^	57.94	57.95	56.22
Public PHC^d^	22.97	18.34	18.89
Private PHC^c^	4.54	4.14	3.62
Pharmacy/drug store, over-the-counter	10.11	14.47	13.00
Public and private hospital	4.44	5.10	8.27
*Current contraceptive methods*			
Pill	24.64	24.30	21.34
Injectables	62.42	59.95	55.24
Implants	5.78	7.06	10.14
IUD	4.65	5.27	8.39
Female sterilization	2.50	3.43	4.88
*Knowledge of modern contraceptive methods*			
knew less than 5 methods	87.98	91.45	90.56
knew 5 methods	12.02	8.55	9.44

^a^Education categories: primary includes elementary school, secondary includes junior and senior high schools, and higher education includes academy, diploma, and university degrees.

^b^Village midwife, midwife, nurse, and FP fieldworker

^c^Clinic, obstetricians, and medical doctor

^d^*Puskesmas*, public Primary Health Care (PHC) clinic and mobile clinic

In 2017, most respondents did not read a newspaper, more than half heard about family planning (FP) from the radio and the television (60.76% and 59.46%), compared to in 2007 and 2012. A majority (87.4%) of the respondents had parity between 0 and 3 children in 2017, a slightly higher percentage than in 2012 (85.8%) and in 2007 (83.72%). Among the sources of modern contraceptives, more than half of the respondents obtained their contraceptives from the midwives, followed by the public primary health care *(Puskesmas)*, 22.97% in 2007 and 18.89% in 2017, and the private PHC combined with the pharmacy/drug store/over the counter (14.65% in 2007 to 18.61% in 2012 and 16.62% in 2017). The public and private hospitals as sources of modern contraceptives were almost doubled in 2017 compared to 2007; as well as the use of implants (10.14%), IUD (8.39%), and female sterilization (4.88%) in 2017, compared to in 2007 (5.78%, 4.65%, and 2.5%, respectively). The injectable remained the highest method used (55.24%) in 2017.

Table 3 shows factors associated with the prevalence of receiving MII information before interaction with time in all three datasets (2007, 2012 and 2017 IDHS). The univariate logistic regression analysis of the 2007 dataset shows that secondary (OR = 2.39, 95% CI 1.51–3.78) and higher education (OR = 5.24, 95% CI 3.14–8.76), compared to no education; being employed as blue-collar (OR = 1.31, 95% CI 1.11–1.55) and white collar workers (OR = 2.34, 95% CI 1.70–3.23) compared to being unemployed; having a wealth quintile of middle (OR = 1.5, 95% CI 1.18–1.93), rich (OR = 2.02, 95% CI 1.60–2.56) and the richest (OR = 2.91, 95% CI 2.30–3.69) compared to the poorest; living in urban areas (OR = 1.43, 95% CI 1.21–1.68) compared to rural areas, significantly increased the odds of receiving MII information. Similarly, hearing about family planning on radio (OR = 1.88, 95% CI 1.53–2.30) compared to not hearing; hearing about family planning on television (OR = 1.72, 95% CI 1.46–2.03) compared to not hearing; and reading about family planning on newspaper (OR = 2.48, 95% CI 2.08–2.96) compared to not reading, significantly increased the odds of receiving MII information. Women with parity equal to four or more (OR = 0.77, 95% CI 0.63–0.95) compared to parity 0–1, showed decreased odds of receiving MII information. Private PHC as a source of family planning method (OR = 1.89, 95% CI 1.37–2.61) compared to midwives increased the odds of receiving MII information, while pharmacy/drug store/over the counter as a source (OR = 0.58, 95% CI 0.44–0.77) compared to midwives decreased the odds of receiving MII information. Currently using IUD (OR = 3.54, 95% CI 2.60–4.83) compared to currently using pills, and knowing all five modern contraceptive methods (OR = 2.05, 95% CI 1.69–2.48) compared to not knowing, increased the odds of receiving MII information.

**Table 3:** Factors associated with the MII information received by reproductive age women using modern contraceptives in Indonesia

	2007 IDHS				2012 IDHS				2017 IDHS			
	OR (SE)	95% CI	AOR (SE)	95% CI	OR (SE)	95% CI	AOR (SE)	95% CI	OR (SE)	95% CI	AOR (SE)	95% CI
*Age*												
15–24 years	1		1		1		1		1		1	
25–34 years	1.17 (0.11)	0.98–1.40	1.07 (0.11)	0.86–1.32	1.4 (0.12)	1.18–1.67	1.31 (0.13)	1.07–1.60	1.27 (0.09)	1.09–1.46	1.21 (0.11)	1.02–1.44
35–49 years	1.14 (0.11)	0.94–1.38	1.07 (0.15)	0.81–1.42	1.33 (0.13)	1.10–1.62	1.32 (0.17)	1.03–1.70	1.15 (0.08)	0.99–1.33	1.23 (0.13)	1.01–1.51
*Education*^*a*^												
No education	1		1		1		1		1		1	
Primary	1.2 (0.27)	0.77–1.88	1.06 (0.24)	0.67–1.67	1.29 (0.29)	0.83–2.02	1.07 (0.25)	0.68–1.70	2.82 (0.91)	1.49–5.31	2.28 (0.75)	1.20–4.34
Secondary	2.39 (0.56)	1.51–3.78	1.70 (0.41)	1.05–2.75	2.23 (0.49)	1.44–3.46	1.56 (0.37)	0.98–2.49	3.95 (1.26)	2.10–7.40	2.69 (0.88)	1.41–5.12
Higher	5.24 (1.37)	3.14–8.76	2.42 (0.70)	1.36–4.29	4.59 (1.05)	2.92–7.21	2.06 (0.56)	1.21–3.50	7.46 (2.43)	3.93–14.16	3.82 (1.30)	1.95–7.48
*Occupation*												
Unemployed	1		1		1		1		1		1	
Agriculture	0.76 (0.07)	0.63–0.93	1.11 (0.12)	0.89–1.38	0.83 (0.09)	0.67–1.02	1.18 (0.13)	0.95–1.47	0.90 (0.07)	0.77–1.07	1.19 (0.11)	0.99–1.43
Blue-collar	1.31 (0.11)	1.11–1.55	1.19 (0.10)	1.00–1.41	1.21 (0.09)	1.05–1.40	1.13 (0.09)	0.97–1.31	1.24 (0.07)	1.11–1.39	1.14 (0.07)	1.02–1.29
White-collar	2.34 (0.38)	1.70–3.23	1.09 (0.20)	0.76–1.57	2.46 (0.30)	1.93–3.14	1.36 (0.21)	1.00–1.85	1.99 (0.19)	1.65–2.41	1.18 (0.14)	0.93–1.48
*Wealth quintile*												
Poorest	1		1		1		1		1		1	
Poor	1.16 (0.14)	0.92–1.46	1.05 (0.12)	0.84–1.33	1.25 (0.12)	1.03–1.51	1.01 (0.1)	0.83–1.24	1.18 (0.09)	1.00–1.39	1.01 (0.09)	0.85–1.21
Middle	1.50 (0.19)	1.18–1.93	1.26 (0.16)	0.98–1.62	1.45 (0.15)	1.18–1.77	1 (0.11)	0.81–1.25	1.33 (0.11)	1.13–1.56	1.05 (0.09)	0.88–1.26
Rich	2.02 (0.24)	1.60–2.56	1.53 (0.21)	1.16–2.02	1.94 (0.19)	1.60–2.35	1.14 (0.13)	0.92–1.42	1.50 (0.12)	1.28–1.76	1.08 (0.10)	0.89–1.30
Richest	2.91 (0.35)	2.30–3.69	1.64 (0.26)	1.20–2.25	2.77 (0.28)	2.27–3.37	1.26 (0.15)	0.99–1.60	2.18 (0.18)	1.84–2.58	1.36 (0.15)	1.09–1.69
*Residence*												
Rural	1		1		1		1		1		1	
Urban	1.43 (0.19)	1.21–1.68	0.89 (0.09)	0.73–1.09	1.54 (0.11)	1.33–1.78	1.11 (0.09)	0.95–1.30	1.29 (0.07)	1.16–1.44	1.08 (0.07)	0.95–1.23
*Region*												
Other Islands	1		1		1		1		1		1	
Java & Bali	1.15 (0.09)	1.00–1.34	1.02 (0.08)	0.87–1.20	1.55 (0.09)	1.36–1.75	1.39 (0.09)	1.21–1.60	1.23 (0.06)	1.11–1.37	1.14 (0.07)	1.01–1.28
*Heard about FP on radio in last few months*												
No	1		1		1		1		1		1	
Yes	1.88 (0.19)	1.53–2.30	1.26 (0.16)	0.99–1.62	2.00 (0.13)	1.75–2.28	1.6 (0.29)	1.13–2.27	1.87 (0.10)	1.68–2.08	1.05 (0.05)	0.66–1.66
*Heard about FP on television in last few months*												
No	1		1		1		1		1		1	
Yes	1.72 (0.14)	1.46–2.03	1.18 (0.12)	0.97–1.44	1.80 (0.11)	1.58–2.05	0.95 (0.16)	0.68–1.31	1.85 (0.09)	1.66–2.05	1.52 (0.35)	0.97–2.39
*Read about FP on newspaper in last few months*												
No	1		1		1		1		1		1	
Yes	2.48 (0.22)	2.08–2.96	1.39 (0.16)	1.11–1.75	2.39 (0.21)	2.00–2.85	1.31 (0.15)	1.06–1.63	2.10 (0.16)	1.80–2.44	1.41 (0.13)	1.18–1.68
*Parity*												
0–1	1		1		1		1		1		1	
2–3	0.98 (0.07)	0.85–1.12	0.95 (0.09)	0.79–1.15	1.08 (0.08)	0.93–1.27	0.98 (0.09)	0.82–1.18	1.03 (0.06)	0.93–1.16	0.97 (0.07)	0.84–1.12
4 +	0.77 (0.08)	0.63–0.95	0.93 (0.13)	0.70–1.23	0.86 (0.09)	0.71–1.05	1.02 (0.13)	0.79–1.32	0.77 (0.06)	0.65–0.90	0.85 (0.09)	0.69–1.05
*Source of FP methods*												
Midwives^b^	1		1		1		1		1		1	
Public PHC^c^	0.90 (0.24)	0.75–1.09	1.02 (0.09)	0.85–1.23	0.97 (0.35)	0.81–1.09	1.09 (0.09)	0.93–1.28	1.01 (0.18)	0.89–1.16	1.00 (0.07)	0.86–1.15
Private PHC^d^	1.89 (0.66)	1.37–2.61	1.13 (0.19)	0.80–1.59	1.52 (0.48)	1.15–2.00	1.11 (0.15)	0.85–1.45	1.41 (0.40)	1.11–1.78	1.04 (0.13)	0.81–1.33
Pharmacy/drug store/over the counter	0.58 (0.34)	0.44–0.77	0.45 (0.07)	0.33–0.61	0.68 (0.40)	0.30–0.57	0.66 (0.08)	0.52–0.83	0.50 (0.21)	0.42–0.61	0.46 (0.06)	0.36–0.60
Hospital	1.18 (0.38)	0.86–1.62	0.88 (0.19)	0.57–1.36	1.25 (0.65)	0.96–1.64	1.02 (0.18)	0.72–1.45	0.94 (0.23)	0.78–1.13	0.66 (0.11)	0.48–0.90
*Current contraceptive methods*												
Pill	1		1		1		1		1		1	
Injectables	0.98 (0.09)	0.82–1.17	0.83 (0.08)	0.67–1.01	1.18 (0.09)	1.01–1.38	1.07 (0.1)	0.89–1.29	1.43 (0.10)	1.25–1.65	0.99 (0.10)	0.82–1.20
Implants	0.91 (0.15)	0.65–1.25	0.76 (0.14)	0.53–1.10	1.24 (0.15)	0.98–1.58	1.12 (0.15)	0.86–1.46	1.54 (0.16)	1.25–1.89	1.12 (0.14)	0.88–1.43
IUD	3.54 (0.56)	2.60–4.83	1.89 (0.35)	1.31–2.74	2.79 (0.39)	2.11–3.69	1.54 (0.25)	1.12–2.11	2.61 (0.26)	2.15–3.18	1.50 (0.20)	1.15–1.95
Female sterilization	1.01 (0.22)	0.66–1.56	0.67 (0.2)	0.37–1.21	1.09 (0.21)	0.74–1.60	0.7 (0.17)	0.43–1.14	1.13 (0.15)	0.86–1.47	0.92 (0.19)	0.61–1.38
*Knowledge of modern contraceptive methods*												
Know < 5 methods	1		1		1		1		1		1	
Know all 5 methods	2.05 (0.20)	1.69–2.48	1.33 (0.14)	1.07–1.64	2.21 (0.21)	1.83–2.67	1.58 (0.17)	1.28–1.94	1.80 (0.14)	1.55–2.10	1.39 (0.11)	1.19–1.63

*AOR* adjusted odds ratio, *CI* confidence interval, *OR* odds ratio, *SE* standard error

^a^Education categories: primary includes elementary school, secondary includes junior and senior high schools, and higher education includes academy, diploma, and university degrees

^b^Village midwife, midwife, nurse, and FP fieldworker

^c^*Puskesmas*, public PHC clinic, mobile clinic

^d^Clinic, obstetricians, and medical doctor

The multivariable analysis of the 2007 dataset in Table 3 revealed that secondary (AOR = 1.70, 95% CI 1.05–2.75) and higher education (AOR = 2.42, 95% CI 1.36–4.29) compared to no education; having a wealth quintile of rich (AOR = 1.53, 95% CI 1.16–2.02) and the richest (AOR = 1.64, 95% CI 1.20–2.25) compared to the poorest; reading about family planning on newspaper (AOR = 1.39, 95% CI 1.11–1.75) compared to not; had significantly higher odds of receiving MII information. But obtaining contraceptive methods from pharmacy/drug store/over the counter (AOR = 0.45, 95% CI 0.33–0.61) compared to midwives, had significantly decreased the odds of receiving MII information. Currently using IUD (AOR = 1.89, 95% CI 1.31–2.74) compared to currently using pills, and knowing all five modern contraceptive methods (AOR = 1.33, 95% CI 1.07–1.64) compared to not knowing all had significantly increased the odds of receiving MII information.

The univariate logistic regression analysis of the 2012 dataset in Table 3 showed that being aged 25–34 years (OR = 1.40, 95% CI 1.18–1.67) and being aged 35–49 years (OR = 1.33, 95% CI 1.10–1.62) compared to being aged 15–24 years, significantly increased the odds of receiving MII information. Having secondary education (OR = 2.23, 95% CI 1.44–3.46) and higher education (OR = 4.59, 95% CI 2.92–7.21) compared to no education; being employed as blue-collar (OR = 1.21, 95% CI 1.05–1.40) and white collar workers (OR = 2.46, 95% CI 1.93–3.14) compared to being unemployed; having a wealth quintile of poor (OR = 1.25, 95% CI 1.03–1.51), middle (OR = 1.45, 95% CI 1.18–1.77), rich (OR = 1.94, 95% CI 1.60–2.35) and the richest (OR = 2.77, 95% CI 2.27–3.37) compared to the poorest; living in urban areas compared to in rural areas (OR = 1.54, 95% CI 1.33–1.78); and living in Java and Bali region (OR = 1.55, 95% CI 1.36–1.75) compared to other islands, significantly increased the odds of receiving MII information. Hearing about family planning on radio (OR = 2.00, 95% CI 1.75–2.28) compared to not; hearing about family planning on television (OR = 1.80, 95% CI 1.58–2.05) compared to not; and reading about family planning on newspaper (OR = 2.39, 95% CI 2.00–2.85) compared to not, significantly increased the odds of receiving MII information. Parity did not associate with the odds of receiving MII information. Private PHC as a source of family planning method (OR = 1.52, 95% CI 1.15–2.00) compared to midwives increased the odds of receiving MII information, while pharmacy/drug store/over the counter as a source of family planning methods (OR = 0.68, 95% CI 0.30–0.57) compared to midwives decreased the odds of receiving MII information. Currently using IUD (OR = 2.79, 95% CI 2.11–3.69) and currently using injectables (OR = 1.18, 95% CI 1.01–1.38) compared to currently using pills; and knowing all five modern contraceptive methods (OR = 2.21, 95% CI 1.83–2.67) compared to not knowing, significantly increased the odds of receiving MII information.

The multivariable analysis of the 2012 dataset in Table 3 revealed that being aged 25–34 years (AOR = 1.31, 95% CI 1.07–1.60) and being aged 35–49 years (AOR = 1.32, 95% CI 1.03–1.70) compared to being young at aged 15–24 years, had significantly higher odds of receiving MII information. Having higher education (AOR = 2.06, 95% CI 1.21–3.50) compared to no education; living in Java and Bali region (AOR = 1.39, 95% CI 1.21–1.60) compared to other islands; hearing about family planning on radio (AOR = 1.60, 95% CI 1.13–2.27) compared to not; and reading about family planning on newspaper (AOR = 1.31, 95% CI 1.06–1.63) compared to not, had significantly higher odds of receiving MII information. Obtaining family planning methods from a pharmacy/drug store/over the counter as a source of family planning methods (AOR = 0.66, 95% CI 0.52–0.83) compared to from midwives was significantly less likely to receive MII information. Currently using IUD (AOR = 1.54, 95% CI 1.12–2.11) compared to currently using pills; and, knowing all five modern contraceptive methods (AOR = 1.58, 95% CI 1.28–1.94) compared to not knowing, had significantly higher odds of receiving MII information.

The univariate logistic regression analysis of the 2017 dataset in Table 3 showed that being aged 25–34 years (OR = 1.27, 95% CI 1.09–1.46) compared to being aged 15–24 years, significantly increased the odds of receiving MII information. Having primary education (OR = 2.82, 95% CI 1.49–5.31), secondary education (OR = 3.95, 95% CI 2.10–7.40) and higher education (OR = 7.46, 95% CI 3.93–14.16) compared to no education; being employed as blue-collar (OR = 1.24, 95% CI 1.11–1.39) and white collar workers (OR = 1.99, 95% CI 1.65–2.41) compared to being unemployed; having a wealth quintile of middle (OR = 1.33, 95% CI 1.13–1.56), rich (OR = 1.50, 95% CI 1.28–1.76) and richest (OR = 2.18, 95% CI 1.84–2.58) compared to the poorest, living in urban areas compared to in rural areas (OR = 1.29, 95% CI 1.16–1.44), and living in Java and Bali region (OR = 1.23, 95% CI 1.11–1.37) compared to other islands, significantly increased the odds of receiving MII information. Hearing about family planning on radio (OR = 1.87, 95% CI 1.68–2.08) compared to not, hearing about family planning on television (OR = 1.85, 95% CI 1.66–2.05) compared to not, and reading about family planning on newspaper (OR = 2.10, 95% CI 1.80–2.44) compared to not, significantly increased the odds of receiving MII information. When parity equal to four or more (OR = 0.77, 95% CI 0.65–0.90) compared to parity 0–1, the odds of receiving MII information decreased. Private PHC as a source of family planning method (OR = 1.41, 95% CI 1.11–1.78) compared to midwives increased the odds of receiving MII information, while pharmacy/drug store/over-the-counter as a source of family planning methods (OR = 0.50, 95% CI 0.42–0.61) compared to midwives decreased the odds of receiving MII information. Currently using injectables (OR = 1.43, 95% CI 1.25–1.65), currently using implants (OR = 1.54, 95% CI 1.25–1.89), currently using IUD (OR = 2.61, 95% CI 2.15–3.18) compared to currently using pills; and, knowing all five modern contraceptive methods (OR = 1.80, 95% CI 1.55–2.10) compared to not knowing, significantly increased the odds of receiving MII information.

The multivariable analysis of the 2017 dataset in Table 3 revealed that being aged 25–34 years (AOR = 1.21, 95% CI 1.02–1.44) and being aged 35–49 years (AOR = 1.23, 95% CI 1.01–1.51) compared to being aged 15–24 years, had significantly higher odds of receiving MII information. Having primary education (AOR = 2.28, 95% CI 1.20–4.34), secondary education (AOR = 2.69, 95% CI 1.41–5.12) and higher education (AOR = 3.82, 95% CI 1.95–7.48) compared to no education; being blue collar AOR = (1.14, 95% CI 1.02–1.29) compared to being unemployed; being richest (AOR = 1.36, 95% CI 1.09–1.69) compared to the poorest; living in Java and Bali region (AOR = 1.14, 95% CI 1.01–1.28) compared to other islands; reading about family planning on newspaper (AOR = 1.41, 95% CI 1.18–1.68) compared to not, had significantly higher odds of receiving MII information. Obtaining family planning methods from a pharmacy/drug store/over the counter (AOR = 0.46, 95% CI 0.36–0.60) and from a public and private hospital (AOR = 0.66, 95% CI 0.48–0.90) compared to midwives were significantly less likely to receive MII information. Currently using IUD (AOR = 1.50, 95% CI 1.15–1.95) compared to currently using pills; and, knowing all five modern contraceptive methods (AOR = 1.39, 95% CI 1.19–1.63) compared to not knowing, had significantly higher odds of receiving MII information.

Results of logistic regression analyses of the MII scores after interaction with ‘time’ (survey year) are shown in Table 4. The 2007 data set was used as the baseline assessment or reference, the 2012 dataset showed the effect of time after 5 years, and the 2017 dataset revealed the effect of time after 10 years. The 2007 baseline showed five variables had statistically significant positive relationships with receiving complete MII information: having reached secondary high school (AOR = 1.7, 95% CI 1.05–2.75) and higher education (AOR = 2.42, 95% CI 1.36–4.29) compared to no education; both being in the ‘rich’ wealth category (AOR = 1.53, 95% CI 1.16–2.02) and the ‘richest’ wealth category (AOR = 1.64, 95% CI 1.20–2.25) compared to the ‘poorest’ wealth category; reading the newspaper (AOR = 1.39, 95% CI 1.11–1.75) compared to not reading; currently using IUD (AOR = 1.89, 95% CI 1.31–2.74) compared to not; and, knew five contraceptive methods (AOR = 1.33, 95% CI 1.07–1.64) compared to not knowing. The pharmacy/drug store/over-the-counter as a source to obtain family planning methods (AOR = 0.45, 95% CI 0.33–0.61) compared to midwives had decreased the odds to receive complete MII information.

**Table 4:** Pattern and determinants of the MII scores (amount information received at the initiation of method use) among women in Indonesia: from 2007 to 2017

	Main effect		Time interaction with MII scores			
	2007 (baseline)		2012 (after 5 years)		2017 (after 10 years)	
	AOR (SE)	95% CI	AOR (SE)	95% CI	AOR (SE)	95% CI
Time			0.60 (0.25)	0.27–1.35	0.49 (0.22)	0.20–1.18
*Age*						
15–24 years	1		1		1	
25–34 years	1.07 (0.12)	0.86–1.32	1.23 (0.19)	0.92–1.65	1.14 (0.16)	0.86–1.49
35–49 years	1.07 (0.16)	0.81–1.42	1.23 (0.24)	0.84–1.80	1.15 (0.20)	0.81–1.63
*Education*^*a*^						
No education	1		1		1	
Primary	1.06 (0.25)	0.67–1.67	1 (0.33)	0.53–1.91	1 (0.87)	0.53–1.91
Secondary	1.70 (0.42)	1.05–2.75	0.90 (0.31)	0.98–4.74	2.16 (0.65)	0.98–4.74
Higher	2.42 (0.70)	1.36–4.29	0.82 (0.33)	0.46–1.75	0.90 (0.71)	0.46–1.75
*Occupation*						
Unemployed	1		1		1	
Agriculture	1.11 (0.12)	0.89–1.38	1.08 (0.17)	0.79–1.47	1.07 (0.16)	0.81–1.43
Blue-collar	1.19 (0.11)	1.00–1.41	0.94 (0.11)	0.75–1.19	0.96 (0.10)	0.78–1.19
White-collar	1.09 (0.20)	0.76–1.57	1.26 (0.31)	0.78–2.02	1.08 (0.24)	0.70–1.66
*Wealth quintile*						
Poorest	1		1		1	
Poor	1.05 (0.12)	0.84–1.33	0.95 (0.15)	0.70–1.29	0.96 (0.14)	0.72–1.28
Middle	1.26 (0.16)	0.98–1.62	0.77 (0.13)	0.56–1.08	0.84 (0.13)	0.61–1.14
Rich	1.53 (0.22)	1.16–2.02	0.72 (0.13)	0.51–1.03	0.72 (0.12)	0.51–1.03
Richest	1.64 (0.26)	1.20–2.25	0.73 (0.15)	0.49–1.09	0.70 (0.16)	0.50–0.99
*Residency*						
Rural	1		1		1	
Urban	0.89 (0.09)	0.73–1.09	1.20 (0.16)	0.93–1.55	1.21 (0.15)	0.95–1.55
*Region*						
Other Islands	1		1		1	
Java & Bali	1.02 (0.08)	0.87–1.20	1.34 (0.15)	1.09–1.66	1.11 (0.11)	0.91–1.36
*Heard about FP on the radio in last few months*						
No	1		1		1	
Yes	1.26 (0.16)	0.99–1.62	1.25 (0.28)	0.81–1.92	0.83 (0.22)	0.49–1.40
*Heard about FP on television in last few months*						
No	1		1		1	
Yes	1.18 (0.12)	0.97–1.44	0.81 (0.16)	0.56–1.19	1.29 (0.32)	0.79–2.12
*Read about FP in the newspaper in last few months*						
No	1		1		1	
Yes	1.39 (0.16)	1.11–1.75	0.94 (0.15)	0.69–1.28	1.01 (0.15)	0.76–1.34
*Parity*						
0–1	1		1		1	
2–3	0.95 (0.09)	0.79–1.15	1.04 (0.14)	0.80–1.34	1.02 (0.12)	0.81–1.29
4 +	0.93 (0.13)	0.70–1.23	1.11 (0.22)	0.76–1.63	0.91 (0.16)	0.65–1.30
*Source of FP methods*						
Midwives^b^	1		1		1	
Public PHC^c^	1.02 (0.35)	0.85–1.23	1.02 (0.19)	0.80–1.31	0.98 (0.20)	0.77–1.24
Private PHC^d^	1.13 (0.56)	0.80–1.59	0.99 (0.23)	0.64–1.53	0.92 (0.25)	0.60–1.40
Pharmacy/drug store/over the counter	0.45 (0.40)	0.33–0.61	1.18 (0.22)	0.74–1.86	1.03 (0.21)	0.69–1.54
Hospital	0.88 (0.46)	0.57–1.36	1.15 (0.34)	0.66–2.02	0.75 (0.22)	0.44–1.28
*Current contraceptive methods*						
Pill	1		1		1	
Injectables	0.83 (0.08)	0.67–1.01	1.43 (0.20)	1.10–1.87	1.2 (0.17)	0.91–1.59
Implants	0.76 (0.14)	0.53–1.10	1.68 (0.37)	1.07–2.63	1.47 (0.33)	0.95–2.29
IUD	1.89 (0.36)	1.31–2.74	0.92 (0.22)	0.57–1.49	0.79 (0.18)	0.50–1.25
Female sterilization	0.67 (0.20)	0.37–1.21	1.18 (0.48)	0.55–2.55	1.37 (0.50)	0.67–2.81
*Knowledge of modern contraceptive*						
Know < 5 methods	1		1		1	
Know 5 methods	1.33 (0.14)	1.07–1.64	1.19 (0.18)	0.89–1.60	0.71 (0.14)	0.81–1.37

*AOR* adjusted odds ratio, *CI* confidence intervals, SE standard error

^a^Primary includes elementary school, secondary includes junior and senior high schools, and higher education includes academy, diploma, and university degrees

^b^Village midwife, midwife, nurse, and FP fieldworker

^c^*Puskesmas*, public Primary Health Care (PHC) clinic, mobile clinic

^d^Clinic, obstetricians, and medical doctor

Table 4 shows after 5 years (2007–2012) only two independent variables still had statistically significant associations with receiving MII complete information: (1) living in the Java Bali region (AOR = 1.34, 95% CI 1.09–1.66) compared to living in other islands, and (2) currently using injectables (AOR = 1.43, 95% CI 1.10–1.87) and currently using implants (AOR = 1.68, 95% CI 1.07–2.63) compared to currently using pills. After 10 years (2007–2017), only one variable (the ‘richest’ in the wealth quintile category (AOR = 0.70, 95% CI 0.50–0.99) compared to the ‘poorest’) still showed a significant association with receipt of complete MII information.

## Discussion

The proportion of Indonesian women who used one of the five modern contraceptives and who received complete MII information increased from 23.85% in 2007 to 28.65% in 2017. However, when survey year is introduced into the analyses the increase in MII over the 10-year period is not statistically significant.

Further, these MII levels are much lower than those seen in other comparable countries in the region. For example, the levels are much lower than those seen in neighbouring countries such as the Philippines (MII scores of 52.5% in 2013) and Cambodia (MII scores of 63.9% in 2010/11) [17]. In addition, as shown in this paper, the increase in MII over the decade between 2007 and 2017 was not statistically significant. In contrast, Cambodia has showed a much larger increase over a shorter period, from 44% in 2005/6 to 63.9% in 2010/11) [17].

In Indonesia, contrary to baseline findings in 2007, 10 years after, the selected socio-demographic characteristics (age, education, occupation, urban–rural residence, living in Java-Bali region or not, exposure of newspaper, television or radio, parity, sources to obtain contraceptives, current method used, or knowledge of five modern contraceptives) were no longer significantly associated with receiving complete MII information. The most likely reasons for the failure to increase the MII over the decade between the 2007 and 2017 surveys lie with changing priorities of the government family planning program as delivered and with increased variability in program performance.

The large population of Indonesia is geographically, culturally and economically diverse. The samples for these surveys were selected to be representative of Indonesia as a whole. Thus, to the extent that the diversity of the country increased between surveys—for example differences between provinces and regions in economic growth, social development and program performance—the greater the variability across the country, the less likely to see significant changes in MII overall. That is, to the extent that the differences between regions and provinces in their social and economic development may increase the variability of the MII results and cause a decrease in the likelihood of statistically significant change over time.

Overall, social programs in Indonesia are still designed to be uniform across the country—one size is assumed to fit all despite great variability between regions in physical, social and economic characteristics—and while priorities within the population program have changed. Currently, the program has shifted its focus into prevention of stunting among children under 2 years old rather than strengthening its focus on improving the use of modern contraceptives. Even so, the content and structure of the population program is similar across the country with the result that there are differences in the effects between regions. The result is that overall program effectiveness is likely to have been reduced.

Thus, the next steps in improving the effectiveness of the program should include analysis at the province and/or regional level with the aim of understanding the extent to which the program outcomes vary between them and the implications of that variation for program content and delivery. Further, with this slow progress Indonesia may not be able to reach the SDG target 3.7.1 that says, “*… to ensure universal access to sexual and reproductive health-care services, including access for family planning, information and education,* by 2030.’’ To reach this goal Indonesia will have to re-focus the family planning program, with emphasis on effective provision of information on contraception through a program adapted in ways that respond to the local issues rather than a uniform program for the whole country with little regard to local geographic, social and economic and human resource differences.

There is plenty of room to improve the focus of the family planning program in Indonesia. It will require a higher priority for family planning activities relative to other activities. Access to the information included in the MII is vital for women to exercise their right to decide freely and responsibly which contraceptives are in accordance with their objective of limiting or spacing their pregnancies. The government needs to increase investments in family planning, especially in the regions where the proportion of women receiving complete MII information is still low. The low MII scores lead to fear of side-effects and other misleading rumours that contribute to discontinuation of contraceptives and eventually increase of unintended pregnancies and bad children’s nutritional status [2].

The family planning program of Indonesia should include the MII scores as an indicator of program performance in promoting modern contraceptives use, which is more reliable than the traditional contraceptives. Jain (2016) stated that receiving adequate information from family planning service providers at the time of contraceptive initiation is essential to help women select a method appropriate for their reproductive health needs and their reproductive goals [19]. Better MII scores will contribute to the continuation of contraceptives use, which in turn will likely increase sustainable contraceptive prevalence. Indonesia performance is still far from the 100% targeted SDGs Indicator 3.7.1 by 2030, but improving the measurable MII scores can gradually improve the quality of information and family planning services as examples from the Philippines and Cambodia show.

## Strengths and limitations

The strength of this study was to use nationally representative data collected using the same methods three times at intervals of 5 years. Regarding limitations, recall bias might occur due to memory lapse. The MII scores were collected among women who used one of the five modern methods (the pill, injectables, implants, IUD and female sterilization) only. Future research should consider including the question about switching, to build a further evidence base for the MII plus values [20] and smaller studies which concentrate on understanding the determinants of contraceptve decision making.

## Conclusions

Findings from this study showed that after 10 years, despite the fact that the MII scores increased gradually across the years, this change was not statistically significant in the 5 years (2007–2012) and in the 10 years (2007–2017) period. Interaction with time did not strengthen the association of any independent variables with higher odds of receiving complete MII information. This means that the gap within the independent variable has closed but there has not been an increase in the informed population between 2007 and 2012. It is recommended that the population program re-focus its efforts on contraception through activities adapted to the local situation especially in those areas with the lowest MII score.

## Data Availability

Data would be made available from the corresponding author on reasonable request.

## Abbreviations

AOR: Adjusted odds ratio
CI: Confidence interval
FP: Family planning
IDHS: Indonesia Demographic and Health Survey
IUD: Intrauterine Devices
MII: Method information index
PHC: Primary Health Care
SE: Standard Errors
SDG: Sustainable Development Goal
